# Internet and Gambling: Insights from Australia’s NBN Rollout

**DOI:** 10.1007/s10899-024-10352-0

**Published:** 2024-08-13

**Authors:** Klaus Ackermann, Sefa Awaworyi Churchill, Musharavati Ephraim Munyanyi

**Affiliations:** 1 Department of Econometrics and Business Statistics, SoDa Labs, Monash Business School Monash University, Melbourne, VIC 3800 Australia; 2 School of Economics, Finance & Marketing, RMIT University, Melbourne, VIC 3000 Australia; 3 Institute for Environmental Economics and World Trade, Leibniz University Hannover, 30167 Hannover, Germany

**Keywords:** Gambling, Internet, Social Capital, Cognitive Function, Australia

## Abstract

Gambling is a well-known leisure activity that leads to significant consequences when consumed excessively. We provide an analysis of the impact of access to faster and more reliable internet connection on gambling. We rely on variations in the rollout of Australia’s largest infrastructure project, National Broadband Network (NBN) installation, to measure internet speed at the postcode level. Using gambling data from the Household, Income and Labour Dynamics in Australia (HILDA) survey, we find that access to high-speed internet is associated with a decline in gambling proxied by the Problem Gambling Severity Index (PGSI). However, a closer look at the various forms of gambling show that internet speed is associated with an increase in online-based gambling activities, which constitute a relatively small proportion of gambling activities that Australians participate in. In contrast, internet speed is associated with a decline in venue-based gambling activities, which constitute a large proportion of gambling activities that occur in Australia, and therefore explains the overall negative effect on gambling. We find that social capital and cognitive functioning are channels through which internet speed influences gambling.

## Introduction

While increased alcohol use, stress, poor social capital, peer pressure and low incomes have been cited as some of the leading causes of gambling (see, e.g., Awaworyi Churchill & Farrell, 2020c; Buchanan et al., 2020; Huggett et al., 2019; Matama et al., 2020; Pitt et al., 2017; Sirola et al., 2019), other studies identify the proliferation of the internet as an important contributor to the epidemic (see, e.g., Effertz et al., 2018; Gainsbury, 2015; Hing et al., 2015; Procter et al., 2019; Wood & Williams, 2011). These studies associate the availability of internet with an increase in gambling rates. This literature demonstrates that the proliferation of the internet has been accompanied by an increase in online and interactive gambling schemes, which have tended to cause a shift from venue gambling toward internet gambling activities (see, e.g., Effertz et al., 2018; Gainsbury et al., 2012; Gainsbury et al., 2015; Hing et al., 2015; Wood & Williams, 2011). Specifically, online gambling has some important advantages that have contributed to increased patronage. For instance, online gambling has been linked with convenience and ease access, anonymity, a wide variety of opportunities and options, lower transaction costs, and instant notification of outcomes (Cotte & Latour, 2009; Gainsbury, 2015). Online gambling has thus been linked with heightened risk of experiencing gambling disorders (see, e.g., Gainsbury et al., 2015; Wu et al., 2015). What are the dynamics and potential implications for gambling behaviour, when internet is significantly faster, suggesting shorter wait times and more prompt access to online services including gaming sites?

This paper attempts to contribute to the internet and gambling literature by examining how access to high-speed internet influences gambling behaviour. Given the benefits of online gambling discussed above, we argue that faster internet connections allow gamblers to access their accounts swiftly and easily, and take advantage of multiple gambling opportunities using different accounts, thus increasing gambling rates. Thus, we examine the impact of high-speed internet access on gambling rates.

We use data from the Household, Income and Labour Dynamics in Australia (HILDA) survey and measure gambling behaviour using the Problem Gambling Severity Index (PGSI), which captures the consumption, consequences and problematic gambling behaviour of respondents (Ferris & Wynne, 2001). We measure internet speed at the Australian postcode level using information on the National Broadband Network (NBN) rollout. The NBN is a nationwide, open-access data network project aimed at replacing the existing analogue telephone service system, which is considered to be relatively slower and less reliable. Considered as the largest nationwide infrastructure project in Australia’s history (Egan, 2008), the NBN is expected to provide significant improvements to the speed of internet connectivity with wired connections providing a minimum of 25 megabit per second (Mbit/s) and up to 1000 Mbit/s (Fitzsimmons, 2013). We construct a new dataset on NBN adoption at the postcode level across Australia as our measure of high-speed internet access. Our econometric strategy takes advantage of the timing of the NBN rollout across Australian postcodes to address the potential endogenous relationship between internet speed and gambling. We find that access to high-speed internet is associated with a decline in gambling. We find that this result is driven by the effect of internet speed on the displacement of venue-based gambling toward online gambling. Specifically, we observe a strong negative effect of internet speed on venue-based gambling activities, which constitute a large proportion of gambling activities that occur in Australia. However, internet speed is associated with an increase in gambling activities that typically occur online.

We make contributions to a least two strands of literature. First, we contribute to the literature that has linked the internet to increased gambling activity (see, e.g., Effertz et al., 2018; Gainsbury, 2015; O’Farrell, 2015; Procter et al., 2019; Wood & Williams, 2011). This literature posits that the internet offers certain advantages (e.g., ease of access, anonymity, convenience and many gambling opportunities), which encourage gambling. We differ from this strand of literature given that we do not focus only on internet use per se but rather how changes in broadband speed influences gambling. We argue that increasing connectivity and the desire to go online, which subsequently leads to exposure to gambling opportunities, are facilitated by internet speed. Thus, people are likely to spend more time online if they have access to fast internet. Understanding the relationship between internet speed and gambling is important given global efforts to promote connectivity via improved internet infrastructure. For instance, besides the ongoing NBN efforts in Australia, efforts have gone into improving internet speeds several other countries including the United States and the United Kingdom (UK) (Cisco, 2020; NBN, 2021; UK Government, 2021). It is important to understand if such upgrades to internet infrastructure are associated with undesirable and detrimental consumption practices. Further, we differ from these studies given that we use longitudinal data from a large nationally representative survey. By using longitudinal data, we are able to trace the gambling patterns of participants, and how they relate to changes in internet speed over time.

Second, we make additions to the general literature that explores the determinants of gambling behaviour (see, e.g., Awaworyi Churchill & Farrell, 2020c; Huggett et al., 2019; Matama et al., 2020; Pitt et al., 2017). Our findings show that in addition to the numerous determinants identified in this literature, including substance use, internet availability, income, and social capital, internet speed is another significant factor that can explain gambling behaviour variations.

## Internet Speed and Gambling

A number of reasons can be proposed for why we can expect internet speed to influence gambling behaviour. In this section, our discussions focus on two main mechanisms – social capital and cognitive functioning – through which internet speed is likely to influence gambling behaviour.

### Social Capital

Social capital can be defined as “the aspects of groups or communities that individuals can draw on when required to provide both mental and material (financial) support” (Awaworyi Churchill & Farrell, 2020c, p.2) or the benefits people derive from their social relationships (Neves, 2013). Some of these aspects and benefits include access to employment opportunities, improved wellbeing, poverty alleviation, better health, access to credit, and civic engagement (see, e.g., Harrison et al., 2019; Huang et al., 2017; Lee et al., 2018; Malual & Mazur, 2017; Nyqvist et al., 2008; Vergani et al., 2021).

The existing literature that has examined the effect of the internet on social capital has yielded conflicting results. For instance, one strand of the literature posits that the internet creates addiction, social isolation and diminishes social capital (see, e.g., Geraci et al., 2018; Sinkkonen et al., 2014). Specifically, exposure to the internet displaces social capital by reducing offline interaction, civic engagement, political participation, and various cultural consumption forms, such as attending theatre shows, concerts and watching movies at cinemas (Geraci et al., 2018). Consequently, there is a higher probability of being socially isolated. This is more so the case for relatively younger people, given that they tend to spend more time on various web-based activities, thus leaving potentially less time for other types of offline leisure activities (Sinkkonen et al., 2014). Further, most internet applications enable users to choose the type of information they need to be exposed to. Such allowances may limit users’ exposure to diverse and useful orientations (Hooghe & Oser, 2015), as users would typically opt for information that reinforces their pre-existing preferences and attitudes (Warner, 2010).

Another strand of the literature associates the internet with increased social capital (see, e.g., Boase & Wellman, 2006; Hooghe & Oser, 2015; Sum et al., 2008; Wang & Wellman, 2010). Findings from this literature suggest that the internet increases social capital by: (1) promoting social connectedness given that the more people connect online, they are likely to eventually transition to face-to-face meetings; (2) encouraging networked individualism, which entails the role of the internet in enabling people to locate a variety of suitable resources and people; (3) allowing people to create their own social networks, which they turn to when they need help; and (4) enabling people to establish social networks larger than those of non-internet users, which allows them to have a large base of resources to draw from when they are in need (Boase & Wellman, 2006). Thus, the internet is argued to create, complement, boost and maintain social capital (Geraci et al., 2018). In this regard, it is expected that with faster internet, social capital would likely be heightened.

The link between social capital and gambling is well-established in the literature. For instance, higher social capital is associated with lower gambling participation (Awaworyi Churchill & Farrell, 2020c). Specifically, given that people tend to indulge in excessive gambling to cope with problems and stresses in their life, individuals who have higher social capital from which to draw resources are unlikely to use gambling as a coping strategy (Awaworyi Churchill & Farrell, 2020c). However, individuals who are socially isolated – which may be due to lack of social capital – are more likely to participate in gambling (McQuade & Gill, 2012; Thomas et al., 2009). Similarly, those with a weak sense of community belonging (i.e., a dimension of cognitive social capital) exhibit higher odds of problem gambling than those with a strong sense of community belonging (Izutsu & Suzuki, 2021).

Relatedly, loneliness – a subjective indicator of social isolation (Holt-Lunstad et al., 2015) – moderates the relationship between gambling problems and daily participation in online gambling communities (Sirola et al., 2019). On the contrary, other evidence indicates that social capital increases gambling participation (Reith & Dobbie, 2011). Here, it is argued that individuals are not born gamblers and do not engage in gambling due to social isolation, but they learn to gamble via their social networks, such as families, friends and communities. Thus the social environment shapes their gambling behaviours. Given the preceding discussion, we hypothesize that:

#### Hypothesis 1

Social capital mediates the relationship between internet speed and gambling behaviour.

### Cognitive Functioning

Cognitive functioning generally refers to a range of mental abilities such as reasoning, attention, learning, problem solving, thinking, memory, decision making, and comprehension. The internet influences cognitive functioning and development in at least three ways. First, online games improve concentration, attention, visual memory and perception, simultaneous processing and speed of processing information (Johnson, 2006). Studies have examined the effect of (online) video-gaming – an interactive, goal-oriented and rule-governed recreational activity – on cognitive functioning (see, e.g., Johnson, 2006; Satyen, 2003; Subrahmanyam, 2001). These studies indicate that video games help improve cognitive processes such as spatial imagery, attention, iconic representation and response time performance (Subrahmanyam, 2001). By compelling players to simultaneously process and perform a wide variety of tasks, such as avoiding hazards and detecting enemies (Johnson, 2006), video gaming enhances players’ visual attention skills (Green & Bavelier, 2003).

Some biological explanations have also been advanced for the effect of the internet on cognitive functioning. For instance, cognitive development relies on environmental stimulation and thus when playing online games, endogenous dopamine, which is a key neurotransmitter responsible for cognitive control (Fukai et al., 2019; Johnson, 2006), is released to enhance cognitive function (Koepp et al., 1998). Given that websites tend to be interactive and contain images and texts that require interpretation, users improve their cognitive functions, including language and literacy, problem solving, concept development and visual information processing (Johnson, 2006), and enhance their metacognitive skills such as searching, planning and evaluation (Gerzog & Haugland, 1999; Tarpley, 2001). Internet usage has therefore been found to enhance the accumulation of cognitive reserve (Ihle et al., 2020), and reduce cognitive decline (Kamin & Lang, 2020; Klimova, 2016; Xavier et al., 2014). Accordingly, we expect that these effects would be made more pronounced by accessing high-speed internet.

High-speed internet, via its effects on cognitive functioning (Ackermann et al., 2023a), may influence gambling. For instance, cognitive abilities such as executive function and premorbid intellectual ability, influence gambling participation. Individuals exhibiting higher premorbid intellectual abilities are less likely to become gamblers (particularly problem gamblers), while those with higher executive function scores (i.e., functions vital for emotional and cognitive self-regulation) (Betancourt et al., 2012) are more likely to engage in gambling (Gong & Zhu, 2019). Evidence shows that pathological gambling is associated with compromised executive cognitive functions, including cognitive control, time estimation, planning tasks, inhibition control and decision making (Goudriaan et al., 2006; Roca et al., 2008).

Pathological gamblers tend to irrationally focus on gambling events and expected outcomes (Se´ vigny & Ladouceur, 2003), and overestimate their perceived control over gambling outcomes, especially when they have a strong desire for the outcomes (Clark, 2014). Such cognitive distortions are also prevalent among all gamblers, including those with good probabilities of winning (Barrault & Varescon, 2013; Mathieu et al., 2018). Specifically, these cognitive distortions influence one’s involvement in gambling activities (Mathieu et al., 2018) and are positively correlated with gambling severity (Miller & Currie, 2008).

Overall, as people are exposed to high-speed internet, we expect significant effects on cognitive function, which is correlated with gambling behaviour. Thus, we set our second hypothesis as follows:

#### Hypothesis 2

Cognitive functioning mediates the relationship between internet speed and gambling behaviour.

## Data and Variables

We use data from two main sources. Data on gambling and associated socioeconomic and demographic characteristics of gamblers are taken from HILDA. HILDA is a national, representative longitudinal survey that tracks Australians. Conducted annually since 2001, HILDA collects information on the dynamics of health, income, work and other socioeconomic aspects of Australians. Our analysis is restricted to waves 15 and 18, corresponding to the years 2015 and 2018, respectively, given that these are the only waves in which gambling data is collected. Data on internet speed, which we merge with HILDA using postcode information, are taken from the Telstra wholesale website.^1^

### Gambling Behaviour

The gambling module in waves 15 and 18 of HILDA includes information on various components of gambling including gambling expenditures, involvement in gambling activities and measures of gambling disorders. Consistent with the literature (see, e.g., Awaworyi Churchill & Farrell, 2020a, b; Baako et al., 2023; Holtgraves, 2008; Loo et al., 2011; Trinh et al., 2022), we use this information to measure the severity of gambling drawing on the PGSI framework (Ferris & Wynne, 2001). The PGSI is a well-established and validated index that has been assessed to be effective in measuring the level of gambling and addiction in population-based samples (Holtgraves, 2008). In HILDA, the PGSI is based on a 9-item questionnaire (shown in Table A1) that captures various dimensions of gambling and the associated consequences experienced in the past 12 months. Responses to the nine items on the questionnaire are on a four-point scale (zero to three), where zero represents ‘never’ and three represents ‘almost always’. The PGSI is a scale that ranges from 0 to 27, with higher scores representing greater gambling and associated problems, and is derived by taking the sum of responses from the items on the questionnaire.

We adopt alternative measures of gambling, which reflect the gambling risk status of participants. The existing literature tends to assign gambling risk categories based on participant PGSI scores (see, e.g., Awaworyi Churchill & Farrell, 2020c). The four risk categories, which are defined as non-problem gamblers, low-risk gamblers, moderate-risk gamblers and problem gamblers, are based on the level of gambling that respondents engage in. Non-problem gamblers have a score of zero on the PGSI scale, and are participants who did not experience any adverse effects from their gambling or did not engage in any problematic gambling behaviour. Low-risk gamblers have PGSI scores of one or two, while moderate-risk gamblers have PGSI scores of three to seven. Problem gamblers have PGSI scores of at least eight and the highest gambling rates with problematic consumption patterns. We derive a four-point gambling scale that reflects the various gambling risk categories, such that one represents ‘non-problem gamblers’ and four represents ‘problem gamblers’. In additional checks, we adopt binary variables that capture each gambling risk spectrum.

We also use the information on gambling expenditures and income to derive an indicator that captures the gambling expenditure share in income. The HILDA survey asks participants: “In a typical month, roughly how much do you spend on the following activities? This includes money spent on-line (on a computer, mobile/smart phone, iPad, etc.).” The response to this question includes a wide range of gambling activities such as scratchies (instant scratch tickets); Bingo (a game of matching numbers in a row); Lotto (Lotto or lottery games); Keno (Lotto-style game played in a casino); private betting (playing cars or mah-jong with friends and family); Poker (card game); casino (casino table games, such as blackjack or roulette); pokies (Poker machines or slot machines); horse or dog races (betting on these races); and sport betting (betting on sports). We use the sum of gambling expenditures across these gambling activities to derive the measure of total gambling expenditure. In a related check, we also use alternative indicators of gambling that are binary variables for each of the gambling activity. These indicators capture whether a participant engages in the specific gambling activity. Furthermore, we use the count of these individual gambling activities as an alternative outcome measure.

Table A2 provides the summary statistics. The highest proportion of gambling activity is playing Lotto with a prevalence of 28% of all respondents in the sample, followed by Poker machines with 8% and scratchies with 7%.

### Internet Speed

Our measure of internet speed is based on the rate of NBN adoption across postcodes in Australia. The NBN is an Australian-wide, open-access data network project that commenced in 2012 to replace to existing internet infrastructure with the aim of improving internet speed and reliability, as well as meet the growing demand for internet in Australia. The project is considered the largest infrastructure development project in Australia’s history (Egan, 2008), and is expected to provide a significant boost to internet connectivity of up to 1000 Mbit/s (Fitzsimmons, 2013).

Consistent with the literature, we construct a dataset on NBN adoption at the postcode level using publicly available information from the Telstra wholesale website (Ackermann et al., 2023a, b). Based on information provided by NBN Co Limited, which is the infrastructure provider, Telstra provides a detailed map of areas covered by the NBN^2^. This information provides details around the areas covered and the respective rollout dates, which we use to calculate the proportion of each postcode that gets upgraded to the NBN at each point in time. Each postcode consists of multiple coverage areas and streets, all of which feature different upgrade times and NBN upgrade completion dates. Accordingly, for a given postcode we observe variation in our measure of internet speed given that some streets or areas have not been upgraded, and for those that have been upgraded, the upgrade completion dates can vary. Our measure of internet speed, which we refer to as ‘share of postcode’, captures the share of each postcode that has been upgraded, and is calculated as the area of a postcode that has been upgraded with NBN as a ratio of the entire postcode area. For instance, for a postcode of size 100 square meters, of which 10 square meters has been upgraded, our measure of internet speed, which reflects NBN rollout, would be 0.1. Given that we focus on gambling measures drawn from waves 15 and 18 of the HILDA survey, we calculate the proportion of postcodes and the time of first upgrade until the last day of 2014 and 2017, respectively. This allows us to examine the impact of internet rollout in the previous period on gambling in the next.

### Covariates

We control for a common set of covariates consistent with the literature on gambling behaviour including gender, age, number of dependents, marital status, disposable household income, employment status, urbanization, educational status, disability status and nationality (see, e.g., Awaworyi Churchill & Farrell, 2018; Delfabbro & Thrupp, 2003; Gainsbury et al., 2012; Tan et al., 2010). In some specification, we also control for personality traits, given the literature that suggests that gambling is influenced by personality (Awaworyi Churchill & Farrell, 2020c; Bagby et al., 2007). We also control for a measure of financial risk taking.

Table A2 provides the summary statistics of all variables used in this study. A first look at the data from the summary statistics suggest that males spend more than twice as much as females on gambling-related activities on average.

### Mediators

We examine social capital and cognitive functioning as potential channels that explain the relationship between internet speed and gambling. To measure social capital, we adopt two measures consistent with the literature (Awaworyi Churchill & Farrell, 2020c; Clark & Lisowski, 2018; Hewitt et al., 2010; Milner et al., 2016). The first measure of social capital (i.e., Social Capital 1), which is based on a 10-item questionnaire reported in Table A4, reflects the dimensions of support that participants perceive they receive from their friends and family. This measure of social capital captures the sentiments of participants on the level of support they believe to get from their friends and families, and is an average of the responses to the items on the questionnaire. The second measure of social capital, which we refer to as Social Capital 2, reflects the level of social contact and interaction with others. This measure of social capital is based on a 10-item questionnaire, which allows participants to assess their level of interactions and social contact with others. The measure of social capital is the average of responses to the items of the scale as shown in Table A5, with higher scores representing higher social capital and vice versa.

HILDA presents information relating to three cognitive functioning tests: (1) a 25-word National Adult Reading Test (NART), (2) Backwards Digit Span (BDS), and (3) Symbol Digits Modalities (SDM). These tests, which are discussed in more detail by Wooden (2013), collectively measure different aspects of cognitive functioning and intelligence. The BDS and SDM tests measure fluid intelligence, which captures problem solving skills and the ability to think or reason in abstract, independent of previous experience or acquired knowledge. The NART test is a measure of crystallized intelligence, which measures the accumulation of skills, knowledge and experience over time (Ashton et al., 2000; Dohmen et al., 2018; Gong & Zhu, 2019; Wooden, 2013). The NART is measured on a 0-to-25-point scale, the BDS on a 0-to-8-point scale, and the SBM, 0 to 100. Higher scores on each cognitive functioning scale represent higher cognitive functioning.

## Methods

We estimate the following model to assess the impact of the NBN rollout on gambling:

1 $${Y_{itp}} = \alpha \> + \beta \>\>NB{N_{tp}} + \gamma {\>^{\prime \>}}X_i^{\prime \>} + \eta {\>_L} + { \in _i}$$

where $\:{Y}_{itL}$, represents the outcome variable, which is the measure of gambling for respondent $\:i$ in at time $\:t$ living in postcode $\:p$; $\:{NBN}_{tp}$ represents the rollout progress of NBN in a given year (i.e., the share of each postcode with NBN access); $\:{X}_{i}$ represents a set of individual level controls variables; $\:\eta\:$ is a local government area (LGA) fixed effect to account for local infrastructure, such as the availability of gambling venues^3^; $\:ϵ$ is an error term. We log transform PGSI to account for the skewness in the data. We cluster the standard errors at the individual level to control for individual specific correlations. For our baseline results, we estimate Eq. (1) using ordinary least squares (OLS) regressions.

However, the underlying relationship between internet speed and gambling risk could be endogenous for many reasons. For instance, people with high gambling patterns or expenditure may self-select into areas that have faster internet connectivity or have more gambling venues to allow for ease of gambling. This could result in reverse causality that may bias our results. Another potential source of endogeneity is omitted variable bias given that it is unlikely to control for all unobservable factors that are likely to be correlated with gambling and internet speed. Thus, we address these concerns by estimation Eq. (1) using the two-stage least squares (2SLS) approach.

Within the 2SLS framework, we instrument for $\:{NBN}_{tp}$ using an instrumental variable (IV) that takes advantage of the variation in timing in which the NBN upgrades are completed across different areas in each postcode. As a principle for identification, for a variable to qualify as an IV in the context of our study: (1) it needs to be correlated with $\:{NBN}_{tp}$ (i.e., share of postcode upgraded onto the NBN) but have no direct correlation with gambling, and (2) the only valid channel through which the IV can influence gambling is via $\:{NBN}_{tp}$. We take advantage of the different NBN rollout dates across postcodes and measure our IV as the number of months since the NBN upgrade commenced in a postcode. The NBN rollout across each postcode happens in stages, with some areas within a postcode getting upgraded first. We use the time in months since the first sub-area in a postcode was connected to the NBN as our IV. Once an area in a postcode gets upgraded, the NBN connection is rolled out across other areas within the postcode over time. Intuitively, the date of first upgrade completion in a postcode sub-area will be correlated with the proportion or share of the entire postcode that gets upgraded over time. The proposed IV is therefore varying across time and postcode.

The fundamental assumption of the validity of our IV rests on the exogenous variation of the NBN upgrade start date, which is uncorrelated with individual gambling. Previous literature has exploited the variation in rollout to instrument for broadband internet access (see, e.g., Bhuller et al., 2013; Campante et al., 2018; Falck et al., 2014).

## Results

Table 1 reports the baseline results for the relationship between internet speed and gambling. Column 1 presents the results for PGSI while Column 2 presents results for the ordinal scale of gambling risk. We find that coefficients on internet speed (or NBN rollout) are negative, but statistically insignificant, suggesting that endogeneity is resulting in downward bias in the baseline estimates. The baseline covariates also suggest some differential trends across demography and socioeconomic factors. Notably, gambling appears to be higher among males compared to females. As expected, higher education seems to have mitigating effects on gambling, and thus, people with higher education are less likely to be at risk of problematic gambling. Higher income is also associated with lower gambling.

**Table 1:** Baseline results

	(1)	(2)
Variables	PGSI	Risk status
Internet speed	-0.004	-0.010
	(0.010)	(0.012)
Male	0.061***	0.073***
	(0.005)	(0.006)
Age	0.000	0.000
	(0.000)	(0.000)
Number of dependents	-0.005*	-0.006*
	(0.003)	(0.003)
Separated	0.036*	0.042*
	(0.019)	(0.023)
Divorced	0.040***	0.047***
	(0.015)	(0.017)
Widowed	-0.010	-0.011
	(0.013)	(0.015)
Single	0.015*	0.019*
	(0.009)	(0.010)
Disposable household income in log	-0.019***	-0.021***
	(0.004)	(0.005)
Employed	0.012*	0.013*
	(0.006)	(0.008)
Living in a major city	-0.001	0.001
	(0.015)	(0.017)
Postgraduate	-0.077***	-0.089***
	(0.009)	(0.010)
Graduate Diploma	-0.052***	-0.060***
	(0.010)	(0.011)
Bachelor	-0.041***	-0.048***
	(0.008)	(0.009)
Diploma	-0.035***	-0.040***
	(0.009)	(0.010)
Certificate	0.007	0.010
	(0.008)	(0.010)
Disabled	0.009	0.010
	(0.007)	(0.008)
Australian	-0.004	-0.004
	(0.007)	(0.008)
Observations	30,665	30,665
R-squared	0.035	0.035
LGA fixed-effect	Yes	Yes

Clustered standard errors at the individual level in parentheses

For relationship status we leave out married as reference, for the education status year 12 and below is the reference and for employment the unemployed category

*** *p* < 0.01, ** *p* < 0.05, * *p* < 0.1

Columns 1 and 2 of Table 2 present 2SLS results for the effects of internet speed on PGSI and the gambling risk spectrum, respectively. Across both columns, the first stage F-statistics are greater than 10 suggesting that at the 10% significance level, our instrumental variables are not weakly correlated with internet speed or the share of a postcode that has access to NBN (Stock & Yogo, 2005). The first stage regression results, which are reported in Appendix Table A3, also show that the effects of our instruments on the share postcode that has access to NBN is consistent with expectations. Specifically, the longer the period since NBN work commenced in a postcode, the higher the share that gains access to NBN.

**Table 2:** 2SLS estimates

	(1)	(2)
Variables	PGSI	Risk status
Internet speed	-0.048**	-0.064**
	(0.023)	(0.027)
	[-0.029]	[-0.033]
Controls included?	Yes	Yes
Observations	30,649	30,649
R-squared	0.033	0.033
LGA fixed-effect	Yes	Yes
Cragg-Donald Wald F statistic	1816	1816

Regressions include covariates consistent with Table 1

Clustered standard errors at the individual level in parentheses

*** *p* < 0.01, ** *p* < 0.05, * *p* < 0.1

The results from Table 2 show that endogeneity causes a significant downward bias in our baseline results since the 2SLS estimates are relatively larger in magnitude and statistically significant compared to the baseline results reported in Table 1. From Column 1, we find that a 1% increase in the share of postcode that gains access to NBN is associated with an average decrease of 0.048% in the PGSI. In terms of standardized coefficients, a one standard deviation increase in NBN coverage or the share of postcode that gains access to NBN is associated with a decrease of 0.029 standard deviations in PGSI. From Column 2, we find that a 1% increase in the share of postcode that gains access to NBN is associated with an average decrease of 0.064 points in gambling measured by the ordinal scale of gambling risk. This represents a 0.033 standard deviation decrease for a one standard deviation increase in faster internet access.

Guided by the gender differences in gambling patterns, we examine the heterogenous effects of internet speed on gambling by gender. Thus, in Table 3, we report the results of internet speed on gambling by gender. Panels A and B report results for the female and male sub-samples, respectively. We find that the coefficients on internet speed is negative across both panels, except for Column 1 of Panel B, where the coefficient is statistically insignificant for the male sub-sample. We probe further into the role of gender in the relationship between internet speed and gambling by incorporating interaction terms between internet speed and gender. The results reported in Panel C of Table 3 confirms that gender moderates the internet speed-gambling relationship. Specifically, we find that the interaction term is statistically significant in both columns. Taking into account the effect size on the other variables in the model, we find that a 1% increase in the share of postcode that gains access to NBN is associated with an average decrease of 0.013 and 0.079% in gambling (PGSI) for males and females, respectively. Similarly, a 1% increase in the share of postcode that gains access to NBN is associated with an average decrease of 0.021 and 0.102 points in gambling (measured by risk status) for males and females, respectively. These results suggest that the effect of internet speed on gambling is more pronounced for females than males.

**Table 3:** Heterogenous effects by gender (2SLS estimates)

	(1)	(2)
Variables	PGSI	Risk status
*Panel A – Female*		
Internet speed	-0.048*	-0.058*
	(0.025)	(0.030)
Observations	16,263	16,263
R-squared	0.036	0.035
LGA fixed-effect	Yes	Yes
Cragg-Donald Wald F statistic	1006	1006
*Panel B – Male*		
Internet speed	-0.052	-0.076*
	(0.039)	(0.046)
Observations	14,338	14,338
R-squared	0.045	0.044
LGA fixed-effect	Yes	Yes
Cragg-Donald Wald F statistic	835.8	835.8
*Panel C – Full sample with interaction*		
Internet speed	-0.079**	-0.102**
	(0.035)	(0.042)
Internet speed*male	0.066*	0.081**
	(0.034)	(0.041)
Male	0.051***	0.061***
	(0.007)	(0.009)
Observations	30,649	30,649
R-squared	0.033	0.033
LGA fixed-effect	Yes	Yes
Cragg-Donald Wald F statistic	1349	1349

Regressions include covariates consistent with Table 1

Clustered standard errors at the individual level in parentheses

*** *p* < 0.01, ** *p* < 0.05, * *p* < 0.1

### Further Analysis

We conduct a series of tests to examine the sensitivity of our results. First, we estimate the effect of internet speed on each gambling risk category as opposed to a single ordinal scale that captures each of the categories. Thus, in Table 4, we report results from a linear probability model that controls for endogeneity where our outcome variables are binary variables set equal to one if an individual falls into a risk category and zero if otherwise. Consistent with our main results, we find that internet speed is associated with an increase in the probability of being a non-problem gambler but a decrease in the probability of being a low-risk gambler. The effect of internet speed on the variables capturing medium-risk and problem gambling are, however, statistically insignificant. The finding here suggests that although internet speed is able to influence gambling behaviour for individuals with no to low gambling expenditures or patterns, it has no significant effect for individuals with problematic gambling patterns. This finding is consistent with the literature on gambling addiction, which suggests that problem gamblers are unlikely to be influenced by socioeconomic factors unless conscious and persistent efforts are made by introducing multiple interventions to change gambling behaviours (see, e.g., Nowak & Aloe, 2014; Pettorruso et al., 2020; Rigbye & Griffiths, 2011; Yip & Potenza, 2014). The finding here also suggests that the effect of internet speed found in Table 2 are likely to be driven by participants with no to low gambling patterns.

**Table 4:** Effects on gambling risk categories (2SLS estimates)

	(1)	(2)	(3)	(4)
Variables	No problem	Low risk	Medium risk	Problem gambler
Internet speed	0.040***	-0.021*	-0.014	-0.005
	(0.015)	(0.011)	(0.009)	(0.006)
Controls included?	Yes	Yes	Yes	Yes
Observations	30,649	30,649	30,649	30,649
R-squared	0.034	0.021	0.022	0.018
LGA fixed-effect	Yes	Yes	Yes	Yes
Cragg-Donald Wald F statistic	1816	1816	1816	1816

Regressions include covariates consistent with Table 1

Clustered standard errors at the individual level in parentheses

*** *p* < 0.01, ** *p* < 0.05, * *p* < 0.1

In a second check, we examine the sensitivity of our results to the control of personality traits. In one of the earliest models of gambling behaviour, Jacobs (1986) suggest that individual differences in personality can explain differences in gambling patterns. We take this into account, and thus control for the Big-Five personality traits in Table 5. In waves 5, 9 and 13 of the HILDA survey, participants are administered questions relating to the Big-Five Personality Inventory. We take advantage of this to control for personality. Consistent with the literature (see, e.g., Gong & Zhu, 2019), we take the average of the personality scores across all waves and include this as an additional control. We also control the level of respondents’ financial risk. To measure financial risk, we take advantage of the response to the HILDA survey question that asks participants to rate the amount of financial risk they are willing to take if they had spare cash. The response to the financial risk question is coded on a four-point scale where one represents ‘takes substantial risks expecting substantial returns’ and four represents ‘not willing to take financial risks’. From Table 5, we find that the effects of internet speed on gambling remain robust with the inclusion of personality and risk in the model. The results also show that while agreeableness has no significant association with gambling, conscientiousness, emotional stability and openness to experience are negatively correlated with gambling. In contrast, extroversion is positively associated with gambling. Consistent with expectations and the literature, unwillingness to take financial risks is associated with a decline in gambling (Awaworyi Churchill & Farrell, 2020a).

**Table 5:** Controlling for the effects of personality and risk level

	(1)	(2)
Variables	PGSI	Risk Status
Internet speed	-0.059***	-0.078***
	(0.023)	(0.027)
Agreeableness	-0.002	-0.002
	(0.004)	(0.005)
Conscientiousness	-0.012***	-0.015***
	(0.003)	(0.004)
Emotional stability	-0.039***	-0.047***
	(0.004)	(0.004)
Extroversion	0.011***	0.012***
	(0.003)	(0.003)
Openness to experience	-0.014***	-0.017***
	(0.003)	(0.004)
Financial risk	-0.081***	-0.093***
	(0.007)	(0.007)
Observations	24,787	24,787
R-squared	0.065	0.063
LGA fixed-effect	Yes	Yes
Controls	Yes	Yes
Cragg-Donald Wald F statistic	1476	1476

Regressions include covariates consistent with Table 1

Clustered standard errors at the individual level in parentheses

*** *p* < 0.01, ** *p* < 0.05, * *p* < 0.1

Overall, our findings suggest that internet speed is associated with lower gambling consumption patterns. This finding contradicts evidence from the literature that suggests the proliferation of internet is an important contributor to higher gambling rates (see, e.g., Effertz et al., 2018; Gainsbury, 2015; Hing et al., 2015; Procter et al., 2019; Wood & Williams, 2011). To probe further and provide further insights into our results, we examine the impact of internet speed on the various types of gambling activities. This analysis is important as it allows us to unravel which activities internet speed influences the most and how these are likely to explain the overall negative effect of internet speed on gambling. Thus, in Table 6 we estimate the impact of internet speed on each gambling activity. On the one hand, we find that internet speed is associated with a lower probability of participating in gambling involving instant scratch cards, Lottery participation and Pokies (or slot machines). On the other hand, we find that internet speed is associating with a higher probability of participating in the various forms of betting and the gambling involving Poker and casino table games.

**Table 6:** Effects by gambling type

	(1)	(2)	(3)	(4)	(5)
Variables	Scratchies	Bingo	Lotto	Keno	Pokies
Internet speed	-0.077***	-0.005	-0.102***	-0.008	-0.057***
	(0.017)	(0.006)	(0.027)	(0.012)	(0.017)
Observations	30,353	30,258	30,414	30,153	30,354
R-squared	0.042	0.033	0.135	0.049	0.049
LGA fixed-effect	Yes	Yes	Yes	Yes	Yes
Controls	Yes	Yes	Yes	Yes	Yes
Cragg-Donald Wald F statistic	1829	1822	1822	1832	1831
	(6)	(7)	(8)	(9)	(10)
	Private betting	Poker	Casino	Horse or dog races	Sport betting
Internet speed	0.024***	0.025***	0.031***	0.008	0.038***
	(0.008)	(0.007)	(0.008)	(0.014)	(0.012)
Observations	30,312	30,295	30,323	30,360	30,308
R-squared	0.018	0.024	0.017	0.055	0.044
LGA fixed-effect	Yes	Yes	Yes	Yes	Yes
Controls	Yes	Yes	Yes	Yes	Yes
Cragg-Donald Wald F statistic	1826	1825	1828	1832	1827

Regressions include covariates consistent with Table 1

Clustered standard errors at the individual level in parentheses

*** *p* < 0.01, ** *p* < 0.05, * *p* < 0.1

The results by gambling type provide some insights into why internet speed is associated with overall lower gambling. It is clear from the gambling type analysis that gambling with instant scratch cards, lotteries and slot machines are driving our results, given that internet speed has a negative effect on these gambling types. These gambling types are typically venue-based or offline gambling activities in Australia. Importantly, these gambling activities also represent the most engaged gambling activities in Australia, besides betting. To put this into context, Lottery participation, instant scratch cards and the use of slot machines represent more than 80% of all the gambling activities in which Australians engage. In terms of gambling expenditure, per capita expenditure on slot machines or electronic gaming machines is highest at $1,292 on average per year (Armstrong & Carroll, 2017). The high per capita expenditure on Pokies or slot machines, which are typically a venue-based or offline gambling activities, has been linked to the fact that Australia has the most electronic gaming machines in the world, which are widely distributed across various outlets including hotels, pubs, casinos and other venues. Specifically, more than 20% of the world’s Pokie machines are in Australia (Lee & Kim, 2014). Overall, the findings here suggest that internet speed has an overall negative effect on gambling by negatively influencing venue-based gambling, and driving gamblers toward online-based gambling, which, in Australia, mostly involves betting. However, betting only represents a small share of the gambling activities in which Australians engage (Armstrong & Carroll, 2017). We also observe positive effects on typical venue-based gambling activities, such as Poker and casino table games^4^. However, these activities collectively only represent 5% of the gambling activities in which Australians engage (Armstrong & Carroll, 2017), and are thus, it is not unexpected that effects on largely consumed activities such as scratch cards and slot machines, which are venue-based will drive the results.

In Table A6, we examine the impact of internet speed on alternative measures of gambling that focus on gambling expenditure and activities count. We find that internet speed is negatively associated with both alternative measures, which is consistent with our main results.

### Social Capital and Cognitive Functioning as Mechanism

Section 2 discussion cognitive functioning and social capital as potential channels through which internet speed influences gambling. For cognitive functioning and social capital to qualify as mechanisms through which internet speed transmits to gambling, in addition to being correlated with internet speed, they should also be correlated with gambling. Further, the inclusion of these variables (i.e., cognitive functioning and social capital) as additional covariates in the regression linking internet speed and gambling should decrease the magnitude of the coefficient on internet speed or render it statistically insignificant (see, e.g., Alesina & Zhuravskaya, 2011; Awaworyi Churchill et al., 2019).

In Table 7, we examine the impact of internet speed on our measures of social capital and cognitive functioning. We find that an increase in internet speed is associated with a decline in both dimensions of social capital. However, internet speed is only significantly associated with NART. Specifically, an increase in internet speed is associated with a decline in crystallized intelligence, which measures the accumulation of skills, knowledge and experience over time. These results suggest that both indicators of social capital and NART are potential mechanisms through which internet speed influences gambling. Thus, we adopt an instrumental variable structural equation model (SEM), in which we include the two indicators of social capital as additional covariates in alternating models that link internet speed with gambling.^5^ In Table 8, the SEM results confirm the negative effect of internet speed on social capital. Additionally, with the inclusion of social capital in the gambling equation, we find that the coefficient on the direct effect of internet speed loses statistically significance. Importantly, the results also show a significant effect of internet speed on gambling is channelled through an indirect effect via social capital. These results confirm that social capital is a channel through which internet speed influences gambling.

**Table 7:** Effects on social capital and cognitive function

	(1)	(2)	(1)	(2)	(3)
Variables	Social Capital 1	Social Capital 2	NART	BDS	SDM
Internet speed	-0.223***	-0.259***	-1.788**	-0.005	1.266
	(0.061)	(0.059)	(0.839)	(0.237)	(1.697)
Observations	30,459	30,490	13,005	13,763	13,054
R-squared	0.094	0.094	0.187	0.073	0.425
LGA fixed-effect	Yes	Yes	Yes	Yes	Yes
Controls	Yes	Yes	Yes	Yes	Yes
Cragg-Donald Wald F statistic	1799	1802	535.3	568.6	536.7

Regressions include covariates consistent with Table 1

Clustered standard errors at the individual level in parentheses

*** *p* < 0.01, ** *p* < 0.05, * *p* < 0.1

**Table 8:** Channel analysis social capital 1

	(1)	(2)	(3)
Variables	Direct	Indirect	Total
*Panel A – Social Capital 1*			
*Equation A – Social Capital*			
Internet speed	-0.055**	N/A	-0.055**
	(0.024)		(0.024)
*Equation B – PGSI*			
Social capital	-0.034***	N/A	-0.034***
	(0.003)		(0.003)
Internet speed	-0.004	0.002**	-0.002
	(0.009)	(0.001)	(0.009)
Observations	30,475	30,475	30,475
Controls	Yes	Yes	Yes
*Panel B – Social Capital 2*			
*Equation A – Social Capital*			
Internet speed	-0.065***	N/A	-0.065***
	(0.023)		(0.023)
*Equation B – PGSI*			
Social capital	-0.033***	N/A	-0.033***
	(0.003)		(0.003)
Internet speed	-0.004	0.002**	-0.002
	(0.009)	(0.001)	(0.009)
Observations	30,506	30,506	30,506
Controls	Yes	Yes	Yes

Regressions include covariates consistent with Table 1

Structural Equation Modelling was used for estimation

Clustered standard errors at the individual level in parentheses

*** *p* < 0.01, ** *p* < 0.05, * *p* < 0.1

## Conclusion

The prevalence of gambling and its associated harms have increased in recent years, and this is more so the case for Australia, which features the highest per capita gambling expenditures in the world. Although several studies have sought to understand the factors that influence gambling, including the availability of internet or internet access, we know nothing about the role of internet speed, and the pathways through which internet speed is likely to influence gambling. This paper thus examined the relationship between internet speed and gambling. We draw on data on Australia’s largest infrastructure project, which has aimed at improving internet speed, to examine if the rollout of NBN or high-speed internet influences gambling. Using data from the HILDA survey, we find that the internet tends to decrease the prevalence of offline or venue-based gambling activities, but increases the prevalence of online-based gambling activities. This finding explains the conclusion that internet speed is associated with an overall decline in gambling given that the bulk of gambling activities that occur in Australia are venue based.

Because many gambling activities occur in social environments and are driven by social factors, we consider social capital as a potential mechanism through which internet speed influences gambling. Additionally, because cognitive abilities, such as premorbid intellectual ability, influence gambling participation, we also examine the role of cognitive functioning as a potential mechanism. We find that by breaking down social capital, internet speed drives individuals away from venue-based gambling activities toward online gambling activities, which do not require much interaction with other individuals. We find evidence of a negative effect of internet speed on cognitive function, specifically crystallized intelligence.

Given that gambling activities are increasingly shifting to online platforms, the findings from this study present reasons for concern. Specifically, although internet speed appears to be driving down venue-based gambling activities, it is associated with an increase in online-based gambling activities. Thus, as more activities move online and are accompanied by a significant shift in the proportion of online activities that people engage in, the availability of high-speed internet would mean an overall increase in gambling. This represents a significant public health concern. From a policy perspective, this evidence suggests the need for policies that ensure proper regulation of online-based gambling activities. Evidence suggests that online gambling activities have not been subjected to the stringent regulations faced by venue-based gambling operators. This poses a significant threat to public health given that with access to high-speed internet, the use of such unregulated gambling activities is likely to increase. Our findings thus speak to the need for a more regulated online gambling sector.

Our study is not without its limitations. Notably, COVID-19 was associated with an increase internet usage (Feldmann et al., 2021), and with that the expectation that online gambling activities will increase. However, given that our data focuses on 2015 and 2018, which pre-date the pandemic, we are not able to explore this dimension. Future studies with relevant data capturing both pre- and post-pandemic can explore this dimension. Another limitation pertains to our inability to compare the pool of people that engage in online gambling before and after the introduction of the NBN. This comparison is important as it could help contextualize our findings given that the proliferation of high-speed internet has been accompanied by several online gambling sites, which people generally do not trust. An important dimension that could offer relevant insights is this comparison coupled with additional analysis focused on the moderating role of online gambling sites. However, we are unable to do this given that we do not have the data to do so.

## Appendices

**Table A1:** Items used in the Problem Gambling Severity Index (PGSI)

	Now thinking about the last 12 months …	Never (0)	Sometimes (1)	Most of the time (2)	Almost always (3)
1	Have you bet more than you could really afford to lose?				
2	Have you needed to gamble with larger amounts of money to get the same feeling of excitement?				
3	When you gambled, did you go back another day to try and win back the money you lost?				
4	Have you borrowed money or sold anything to get money to gamble?				
5	Have you felt that you might have a problem with gambling?				
6	Has gambling caused you any health problems, including stress or anxiety?				
7	Have people criticized your betting or told you that you had a gambling problem, regardless of whether or not you thought it was true?				
8	Has your gambling caused any financial problems for you or your household?				
9	Have you felt guilty about the way you gamble or what happens when you gamble?				

**Table A2:** Summary statistics

Variable	All	Female	Male
NBN proportion of the postcode	0.15 (0.24)	0.15 (0.24)	0.15 (0.24)
Month since NBN worked commenced	31.87 (28.11)	31.67 (27.76)	32.10 (28.50)
Problem Gambling Severity Index (PGSI)	0.28 (1.51)	0.19 (1.19)	0.39 (1.80)
Risk status	1.12 (0.46)	1.08 (0.38)	1.16 (0.54)
Non problem gambler	0.93 (0.26)	0.95 (0.22)	0.90 (0.30)
Low risk gambler	0.04 (0.19)	0.03 (0.17)	0.05 (0.22)
Moderate risk gambler	0.02 (0.15)	0.02 (0.13)	0.03 (0.18)
Problem gambler	0.01 (0.10)	0.01 (0.08)	0.01 (0.12)
Gambling related expenditure (in $000s)	0.53 (5.88)	0.33 (1.82)	0.77 (8.37)
Male	0.47 (0.50)	0.00 (0.00)	1.00 (0.00)
Age	45.87 (19.02)	46.08 (19.09)	45.63 (18.94)
Number of dependents	0.58 (1.01)	0.60 (1.01)	0.56 (1.00)
Separated	0.03 (0.16)	0.03 (0.16)	0.02 (0.15)
Divorced	0.06 (0.24)	0.07 (0.26)	0.05 (0.21)
Widowed	0.05 (0.21)	0.07 (0.25)	0.02 (0.14)
Single	0.23 (0.42)	0.21 (0.41)	0.24 (0.43)
Married	0.48 (0.50)	0.46 (0.50)	0.50 (0.50)
Disposable household income (in $000s)	98.93 (68.83)	96.81 (68.79)	101.34 (68.81)
Employed	0.63 (0.48)	0.59 (0.49)	0.68 (0.47)
Unemployed	0.04 (0.20)	0.04 (0.19)	0.04 (0.21)
Not in the labour force	0.33 (0.47)	0.37 (0.48)	0.28 (0.45)
Living in a major city	0.62 (0.48)	0.62 (0.49)	0.62 (0.48)
Postgraduate	0.06 (0.23)	0.06 (0.23)	0.06 (0.23)
Graduate Diploma	0.06 (0.24)	0.07 (0.25)	0.05 (0.22)
Bachelor	0.15 (0.36)	0.16 (0.37)	0.13 (0.34)
Diploma	0.10 (0.30)	0.10 (0.30)	0.09 (0.29)
Certificate	0.23 (0.42)	0.18 (0.38)	0.28 (0.45)
Disabled	0.24 (0.43)	0.24 (0.43)	0.23 (0.42)
Australian	0.79 (0.40)	0.80 (0.40)	0.79 (0.41)
Agreeableness	5.39 (0.83)	5.61 (0.77)	5.13 (0.82)
Conscientiousness	5.06 (0.93)	5.15 (0.94)	4.96 (0.91)
Emotional stability	5.16 (0.96)	5.17 (0.99)	5.15 (0.93)
Extroversion	4.44 (1.00)	4.54 (1.04)	4.32 (0.94)
Openness to experience	4.22 (0.97)	4.18 (0.99)	4.26 (0.95)
Financial risk	3.42 (0.68)	3.54 (0.61)	3.29 (0.73)

*Note* Standard deviation in brackets

**Table A3:** First stage regression

	(1)
Variables	First stage
Months since NBN work commenced in postcode	0.0046*** (0.0001)
Male	-0.0025 (0.0026)
Age	0.0004*** (0.0001)
Dependents	0.0007 (0.0014)
Separated	0.0127 (0.0083)
Divorced	0.0089 (0.0056)
Widowed	0.0061 (0.0068)
Single	0.0145*** (0.0040)
Income	0.0042** (0.0021)
Employed	-0.0011 (0.0032)
Living in a major city	0.0533*** (0.0069)
Postgraduate	0.0091 (0.0060)
Graduate Diploma	0.0062 (0.0058)
Bachelor	0.0081** (0.0041)
Diploma	-0.0024 (0.0046)
Certificate	0.0014 (0.0034)
Disabled	-0.0083** (0.0034)
Australian	0.0044 (0.0034)
Observations	30,665
R-squared	0.4037
LGA fixed-effect	Yes
F-statistics	420.4

Standard errors in parentheses

*** *p* < 0.01, ** *p* < 0.05, * *p* < 0.1

**Table A4:** Items used in the Social Capital 1 Index

	The following statements have been used by many people to describe how much support they get from other people. How much do you agree or disagree with each?…
1	I enjoy the time I spend with the people that are important to me
2	I seem to have a lot of friends
3	There is someone who can always cheer me up when I am down
4	When I need someone to help me out, I can usually find someone
5	When something’s on my mind, just talking with the people I know can make me feel better
6	People don’t come and visit as much as I would like (reverse coded)
7	I often need help from other people but can’t get it (reverse coded)
8	I don’t have anyone that I can confide in (reverse coded)
9	I have no one to lean on in times of trouble (reverse coded)
10	I often feel very lonely (reverse coded)

*Note* Response scale is: strongly disagree (1) … strongly agree (7)

**Table A5:** Items used in the Social Capital 2 Index

	The following statements have been used by many people to describe how much support they get from other people. How much do you agree or disagree with each?…
1	People don’t come to visit me as often as I would like (reverse coded)
2	I often need help from other people but can’t get it (reverse coded)
3	I seem to have a lot of friends
4	I don’t have anyone that I can confide in (reverse coded)
5	I have no one to lean on in times of trouble (reverse coded)
6	There is someone who can always cheer me up when I’m down
7	I often feel very lonely (reverse coded)
8	I enjoy the time I spend with the people who are important to me
9	When something’s on my mind, just talking with the people I know can make me feel better
10	When I need someone to help me out, I can usually find someone

*Note* Response scale is: strongly disagree (1) … strongly agree (7)

**Table A6:** Alternative outcome measures

	(1)	(2)
Variables	Expenditure	Activities
Internet speed	-0.001*	-0.119*
	(0.001)	(0.071)
Observations	30,455	30,649
R-squared	0.008	0.074
LGA fixed-effect	Yes	Yes
Cragg-Donald Wald F statistic	1807	1816
Controls	Yes	Yes

Regressions include covariates consistent with Table 1

Clustered standard errors at the individual level in parentheses

*** *p* < 0.01, ** *p* < 0.05, * *p* < 0.1

## Footnotes

1 https://www.telstrawholesale.com.au/nbn/nbn-rollout-schedule.html last accessed 4 January 2021.

2 https://data.gov.au/dataset/ds-dga-ed264f72-5fdf-4a54-8381-d35a10c7c85a/details?q=statutory last accessed 5 January 2021.

3 In Australia, an LGA is made up of several postcodes.

4 There is also a growing shift in gambling products and thus poker and casino table games are increasingly becoming online games (St-Pierre et al., 2014).

5 Given that data for cognitive function is only available for selected waves that do not match the waves for which gambling data is available, we do not proceed with this second stage analysis using the NART. Thus, the evidence on NART as a channel is only suggestive and should be taken with caution.
